# Elevated serum inflammasome adaptor protein ASC is associated with white matter hyperintensities in vascular cognitive impairment

**DOI:** 10.1093/braincomms/fcag068

**Published:** 2026-03-05

**Authors:** Yuek Ling Chai, Saima Hilal, Vera Yuan Cai, Vincent C T Mok, Joyce R Chong, Yingqi Liao, Boon Yeow Tan, Narayanaswamy Venketasubramanian, Thiruma V Arumugam, Christopher Li-Hsian Chen, Mitchell K P Lai

**Affiliations:** Department of Pharmacology, Yong Loo Lin School of Medicine, National University of Singapore, Singapore 117600, Singapore; Memory, Aging and Cognition Centre, National University Health System, Singapore 117599, Singapore; Department of Pharmacology, Yong Loo Lin School of Medicine, National University of Singapore, Singapore 117600, Singapore; Saw Swee Hock School of Public Health, National University of Singapore, Singapore 117549, Singapore; Gerald Choa Neuroscience Institute, Li Ka Shing Institute of Health Sciences, Lau Tat-Chuen Research Centre of Brain Degenerative Diseases in Chinese, Division of Neurology, Department of Medicine and Therapeutics, Faculty of Medicine, The Chinese University of Hong Kong, Hong Kong SAR 999077, China; Gerald Choa Neuroscience Institute, Li Ka Shing Institute of Health Sciences, Lau Tat-Chuen Research Centre of Brain Degenerative Diseases in Chinese, Division of Neurology, Department of Medicine and Therapeutics, Faculty of Medicine, The Chinese University of Hong Kong, Hong Kong SAR 999077, China; Department of Pharmacology, Yong Loo Lin School of Medicine, National University of Singapore, Singapore 117600, Singapore; Memory, Aging and Cognition Centre, National University Health System, Singapore 117599, Singapore; Department of Pharmacology, Yong Loo Lin School of Medicine, National University of Singapore, Singapore 117600, Singapore; Memory, Aging and Cognition Centre, National University Health System, Singapore 117599, Singapore; St Luke’s Hospital, Singapore 659674, Singapore; Raffles Neuroscience Centre, Raffles Hospital, Singapore 188770, Singapore; La Trobe Institute for Molecular Science, Department of Microbiology, Anatomy, Physiology and Pharmacology, School of Agriculture, Biomedicine and Environment, La Trobe University, Bundoora , Victoria 3086, Australia; Department of Pharmacology, Yong Loo Lin School of Medicine, National University of Singapore, Singapore 117600, Singapore; Memory, Aging and Cognition Centre, National University Health System, Singapore 117599, Singapore; Department of Pharmacology, Yong Loo Lin School of Medicine, National University of Singapore, Singapore 117600, Singapore; Memory, Aging and Cognition Centre, National University Health System, Singapore 117599, Singapore

**Keywords:** biomarkers, cerebral vascular diseases, inflammasomes, vascular cognitive impairment, white matter hyperintensities

## Abstract

Alzheimer’s disease (AD) and vascular dementia (VaD) exhibit distinct neuropathological hallmarks, with AD defined by cortical amyloid plaques and neurofibrillary tangles, while VaD arises primarily from cerebrovascular disease (CeVD). However, CeVD frequently coexists with AD, and both conditions fall within the spectrum of vascular cognitive impairment (VCI). Notably, chronic neuroinflammation is a shared mechanistic link between AD and VaD. Inflammasomes, multi-protein complexes that sense cellular damage and orchestrate inflammatory responses, have been implicated in preclinical models of both diseases. However, their translational potential in the clinical setting remains underexplored. Here, we investigate the inflammasome adaptor protein, apoptosis associated speck-like protein containing a CARD (ASC), as a novel serum biomarker for CeVD, cognitive decline and VCI progression. A total of 531 participants (123 non-cognitively impaired, 208 cognitive impaired, no dementia and 200 with dementia) were included from a Singapore-based clinical cohort. All subjects underwent comprehensive clinical, neuropsychological and neuroimaging assessments. Serum samples were collected, and ASC was measured using a microfluidics immunoassay platform. Compared to controls, serum ASC levels were increased in the VCI clinical subgroups, namely, cognitive impaired, no dementia with CeVD, AD with CeVD and VaD. Multivariate analyses using CeVD neuroimaging markers showed associations between serum ASC and white matter hyperintensities, but not with lacunes or cerebral microbleeds. Mediation analyses similarly showed that the association between ASC and VCI subgroups may be largely explainable by severity of concomitant CeVD, specifically white matter hyperintensities volume. Our findings indicate that elevated serum ASC levels may reflect a state of inflammasome activation in VCI, particularly in individuals with extensive white matter hyperintensities. These results underscore the potential role of dysregulated inflammasome activation not only as a pathogenic factor but also as a potential therapeutic target for VCI.

## Teaser

Chai *et al*. report that higher serum inflammasome adaptor protein ASC were associated with vascular cognitive impairment and dementia, which may be largely mediated by concomitant cerebrovascular disease, specifically white matter hyperintensities. Their findings suggest the roles of dysregulated inflammasome as pathogenic factor and therapeutic target for vascular cognitive impairment.

## Graphical Abstract

**Graphical Abstract:**
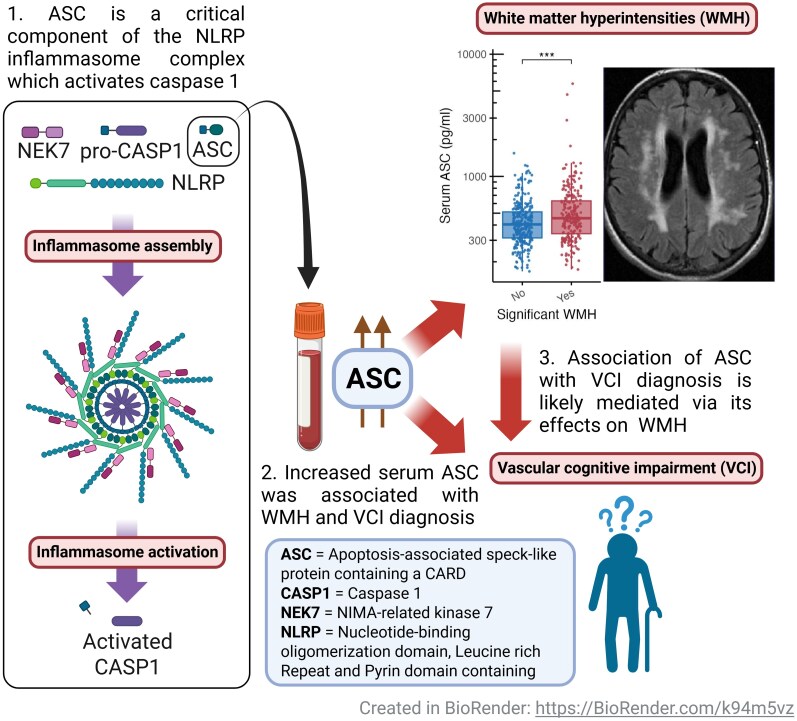

## Introduction

Alzheimer’s disease (AD) and vascular dementia (VaD) are the two primary causes of dementia in the elderly. The pathophysiological mechanisms underlying cognitive impairment and dementia are complex and heterogeneous, and while AD and VaD represent disparate disease aetiologies involving cortical amyloid plaques/neurofibrillary tangles and cerebrovascular disease (CeVD), respectively, these pathologies often coexist, thus prompting the use of the term, ‘vascular cognitive impairment (VCI)’, to describe the full spectrum of cognitive symptoms caused by CeVD, ranging from mild cognitive impairment to VaD, either in isolation or in combination with AD-associated neurodegeneration.^1^

Chronic neuroinflammation is increasingly recognized as a fundamental pathological hallmark of both AD and VCI, contributing to disease progression through sustained immune activation and neurovascular dysfunction.^2,3^ In AD, Aβ species comprising 40–42 amino acids have been shown to trigger microglial activation, leading to the release of pro-inflammatory cytokines, including interleukin (IL)-1β, IL-6, and tumour necrosis factor (TNF), thereby exacerbating neuroinflammation and contributing to disease progression.^4-6^ The initial ability of these acute inflammatory responses to clear aggregated Aβ diminishes as amyloid fibrils accumulate, leading to dysfunctional, chronic neuroinflammation, unresolved cytokine secretion, subsequent synaptic dystrophy and eventual neuronal cell death underlying the cognitive deficits of AD.^7-9^ On the other hand, activated glial cells are also found to colocalize around cortical vascular lesions, together with elevated inflammatory markers in brain and blood of people with VCI.^10-12^ While the exact mechanisms are not fully understood, animal studies suggest chronic cerebral hypoperfusion (CCH) as the main driver of a vicious cycle between exacerbating inflammation and endothelial damage, leading to progression of CeVD, especially white matter lesions, and eventually to neuronal loss associated with learning deficits.^13-16^ Taken together, there is now established preclinical and clinical evidence for the pathogenic role of dysregulated neuroinflammation in AD and VCI. However, neuroinflammation is a complex phenomenon involving multiple cell types, signalling pathways and interacting molecules, many of which have not yet been well-characterized. In this study, we focused on the potential involvement of inflammasomes in AD and VCI.

Inflammasomes are intracellular multi-protein complexes which play important roles in detecting cellular damage and mediating inflammatory responses to pathogens and disease processes.^17^ There are four canonical inflammasome sensors [absent in melanoma 2, AIM2; nucleotide-binding oligomerization domain-like receptor protein 1 and 3, NLRP1, NLRP3; and NOD-like receptor family caspase activation and recruitment domain (CARD)-containing protein 4, NLRC4], pattern recognition receptors which can detect exogenous pathogens, endogenous injury or danger signals and cellular stress.^16^ Activation of these receptors leads to inflammasome assembly involving the universal adaptor protein, apoptosis associated speck-like protein containing a CARD (ASC), in turn leading to caspase-1 activation along with downstream cytokine secretion and inflammatory responses.^17,18^ Previous clinical studies showed ASC upregulation in patients with mild cognitive impairment and AD.^19,20^ In contrast, relatively little is known about the involvement of inflammasomes in VCI, despite ample preclinical evidence of dysregulated inflammasome activation in animal models of CCH.^21-23^ Furthermore, it is unclear whether peripheral markers of inflammasome activation may be associated with CeVD neuroimaging measures.

This study aimed to characterize serum ASC levels in a Singapore-based VCI cohort and to systematically evaluate its associations with quantitative neuroimaging markers of CeVD, including white matter lesions, lacunes and cerebral microbleeds, to elucidate its potential role as a biomarker for cerebrovascular pathology in cognitive impairment.

## Methods

### Ethics approval and consent to participate

Institutional Review Board approval for the study was obtained from the Singapore National Healthcare Group Domain-Specific Review Board (reference 2010/00017; study protocol DEM4233).

### Clinical study participants

This cross-sectional study enrolled participants with cognitive impairment no dementia (CIND) and participants with dementia, who were recruited from August 2010 to July 2015 at two sites in Singapore: the National University Hospital and Saint Luke’s Hospital. Control participants—defined as having normal cognitive function based on objective neuropsychological testing—were recruited from memory clinics as well as from the community^24^ (see Supplementary Fig. 1 for participant recruitment flowchart). The study was approved by the National Healthcare Group Domain-Specific Review Board in Singapore (reference 2010/00017; protocol DEM4233) and conducted in accordance with the Declaration of Helsinki. Written informed consent was obtained from all participants or their legally authorized caregivers in their preferred language before enrolment. Participants underwent standard physical examinations, clinical assessments, blood tests, neuropsychological evaluations, and neuroimaging at the National University of Singapore. Detailed study procedures have been described in previous publications.^25^

### Clinical assessments and diagnoses

Cognitive impairment and dementia diagnoses were determined during weekly consensus meetings attended by study clinicians and neuropsychologists. CIND was diagnosed based on clinical judgment and published protocol,^25^ namely, impairment in at least one domain of the neuropsychological test battery (see below section on Neuropsychological assessments) without significant loss of independence in daily living. Dementia was diagnosed based on the Diagnostic and Statistical Manual of Mental Disorders, 4th edition (DSM-IV) criteria, with further classification into AD or VaD based on clinical consensus criteria [the National Institute of Neurological and Communicative Disorders and Stroke and the Alzheimer's Disease and Related Disorders Association (NINCDS-ADRDA) criteria for AD,^26^ and the National Institute of Neurological Disorders and Stroke-Association Internationale pour la Recherché et l’ Enseignement en Neuroscience (NINDS-AIREN) criteria for VaD^27^]. Additionally, plasma tau phosphorylated at threonine 217 (p-tau217) concentrations as a validated biomarker for brain amyloid were measured using the SIMOA platform as previously described.^28^

### Neuropsychological assessments

All participants underwent cognitive tests, including the Mini-Mental State Examination (MMSE), the Montreal Cognitive Assessment (MoCA) and a locally validated, detailed neuropsychological test battery,^29^ administered by trained research psychologists. The test battery assessed seven cognitive domains, namely, Executive Function, Attention, Language, Visuomotor Speed, Visuoconstruction, Visual Memory and Verbal Memory (see Supplementary Table 1 for a summary of the component tests for each domain).

### Assessments of other risk factors

Risk factors including hypertension, hyperlipidaemia, diabetes mellitus and cardiovascular disease were identified through clinical interviews and review of medical records and were recorded as either present or absent. Further details on these ascertainment methods are provided in a previous publication.^24^ Educational attainment was dichotomized as low (limited to primary school or less) or high (more than primary school). Apolipoprotein E (APOE) genotyping was conducted using methods reported previously,^30^ and participants were classified as APOE ε4 carriers if they possessed at least one ε4 allele.

### Neuroimaging markers of CeVD

Magnetic resonance imaging (MRI) scans were performed on a 3-Tesla Siemens Magnetom Trio Tim scanner, using a 32-channel head coil, at the Clinical Imaging Research Centre, National University of Singapore. The majority (92.7%) of the MRI assessments was done < 1 year before or after blood collection (median & 11 months). Participants were excluded if they had claustrophobia, any contraindications to MRI, or were unable to tolerate the scanning procedure. All MRI scans were independently graded by one radiologist and two clinicians who were blinded to the participants’ neuropsychological test results and clinical information. The sequences included T1-weighted Magnetization Prepared Rapid Gradient Recalled Echo (MPRAGE), Fluid Attenuated Inversion Recovery (FLAIR), T2-weighted and Susceptibility Weighted Imaging (SWI) sequences for the measurement of the following CeVD markers: (i) white matter hyperintensities (WMH, a marker for white matter lesions) were graded using the Age-Related White Matter Changes scale (ARWMC)^31^; (ii) quantitative WMH volumes expressed as percentage of total intracranial volumes were determined using AccuBrain® IV 2.0 (BrainNow Medical Technology Ltd, Hong Kong SAR, China), a fully automated software for brain volumetric segmentation and quantification^32^; (iii) lacunar and cortical infarcts were defined on FLAIR and T2 sequences using the STRIVE criteria^33^; and (iv) cerebral microbleeds (CMBs) were defined on SWI sequences using the Brain Observer Microbleeds Anatomical Rating Scale.^34^ Significant CeVD was defined as the presence of cortical infarcts and/or ≥2 lacunes and/or confluent WMH (ARWMC score ≥ 8) in two regions of the brain, as described previously.^24^

### Serum ASC measurements

Non-fasting blood samples were collected from participants into serum-separating tubes and centrifuged at 2000*g* for 10 min at 4°C. The resulting serum supernatant was carefully aliquoted and stored at −80°C until analysis. All blood measurements were performed in duplicate, with laboratory personnel blinded to participants’ clinical and demographic characteristics. ASC concentrations (in pg/mL) were measured using an automated quantitative microfluidics immunoassay platform (Ella ProteinSimple™, Bio-techne, Minneapolis, MN, USA) following manufacturer’s instructions. Briefly, serum samples were centrifuged at 16 000 *g* at 4°C for 20 min. Supernatants of each sample were extracted and diluted 2-fold in diluent buffer before adding to the sample wells on Ella cartridges, loading onto the cartridge holder and commencing the automated immunoassay process. A standard curve ranging from 5.24 to 8000 pg/mL was generated for each assay and fitted to a five-parameter logistic model. Sample concentrations read from the standard curve were multiplied by the dilution factor of 2 to obtain the actual ASC levels in serum.

### Statistical analyses

Statistical analyses were conducted using SPSS Statistics (version 21; IBM Corp., Chicago, IL, USA), R (version 3.6.3; The R Foundation) and RStudio (version 1.2; RStudio, Inc., Boston, MA, USA). Analysis of variance (ANOVA) and *χ*² tests were applied to compare baseline characteristics between the participant groups with and without the condition (cases and controls). Given that serum ASC concentrations were not normally distributed (Shapiro–Wilk test *P* < 0.001, skewness = 8.02, kurtosis = 93.9), ASC levels were log-10 transformed and included as a determinant, whereas diagnostic groups stratified by CeVD status were defined as outcomes. The R ‘brglm2’ package^35^ was used to perform multinomial regression analyses using bias reduction method. Effect measures were expressed in odds ratios (OR) and 95% confidence intervals (CI). Unadjusted model was first assessed (Model I), followed by adjustment for demographic characteristics including age, gender, education and APOE ε4 (Model II), and lastly with additional adjustment for vascular risk factors including hypertension and diabetes (Model III), as these variables were not matched between the diagnostic groups (see Supplementary Table 2).

To examine possible associations between ASC and CeVD, regression models were performed with log-transformed ASC included as a determinant and specific MRI markers of CeVD defined as outcomes. Generalized linear models with Gaussian distribution were performed using R base ‘glm’ function for WMH visual grading based on ARWMC scores, as well as for quantitative WMH volume (expressed as percentage of total intracranial volume), with the associations with log-transformed ASC expressed in mean differences (B) and 95% CI. Because CMBs and lacunar infarcts were rarely detected in non-VCI subgroups, zero-inflated negative binomial regression models were constructed using R package ‘pscl’ for the count of lacunar infarct and CMB, with the associations with log-transformed ASC expressed in rate ratios (RR) and 95% CI.^36,37^ All models were adjusted for age, gender and hypertension as these variables were not matched between groups (see Supplementary Table 3). Sensitivity analyses were repeated to include only cognitively impaired subjects, i.e. CIND and dementia. Furthermore, causal mediation analyses to assess CeVD as mediators of the associations between ASC and VCI were performed using R package ‘mediation’ to estimate the effects and mediation proportion based on probability scale.^38^ To obtain effect estimates of each path in log-odd scale, R base ‘glm’ function was used to construct the generalized linear models for each path, followed by R base ‘coef’ function to extract the effect estimates in log-odds scale. Bootstrap percentile method with 5000 simulations using R package ‘boot’ were then performed to estimate the corresponding *P* values.^39^ All models were adjusting for age, gender, education, APOE ε4, hypertension and diabetes. All tests were two-sided and *P* < 0.05 were considered statistically significant.

## Results

### Characteristics of study participants

Of the 531 subjects included in the study, 123 had no cognitive impairment (NCI), 208 were cognitively impaired, no dementia (CIND) and 200 had dementia (155 AD and 45 VaD). Table 1 shows the cross-sectional demographic and disease characteristics of the study cohort, with cases (CIND and/or dementia) being significantly older, having fewer years of education and higher prevalence of APOE ε4, hypertension and diabetes compared to NCI. Notably, while the diagnoses of AD and VaD patients were based on clinical criteria, they showed distinct biomarker profiles consistent with their disease status. Specifically, plasma p-tau217, a validated marker of brain amyloid burden,^28^ was significantly increased in AD but not in VaD (55.7% in AD and 9.4% in VaD), when compared to NCI as well as CIND groups (Pearson’s *χ*² with Bonferroni’s *post hoc* test, *P* < 0.001; Supplementary Fig. 2).

**Table 1:** Cross-sectional demographic and disease characteristics of study cohort

	NCI	CIND	Dementia	*P* value
*n*	123	208	200	
Age, years, mean (SD)	69.5 (7.5)	74.1 (7.4) a	75.9 (7.9) a	**<0**.001*
Female, *n* (%)	68 (55.3)	105 (50.5)	119 (59.5)	0.187^†^
Education ≤ elementary, *n* (%)	39 (31.7)	94 (45.2) b	143 (71.5) b,c	**<0**.**001^†^**
Hypertension, *n* (%)	77 (62.6)	143 (69.4)	158 (79.4) b	**0**.**003^†^**
Diabetes, *n* (%)	29 (23.6)	69 (33.2)	82 (41.0) b	**0**.**006^†^**
Hyperlipidaemia, *n* (%)	87 (70.7)	158 (76.3)	150 (75.0)	0.519^†^
Heart disease, *n* (%)	8 (6.6)	31 (14.9)	23 (11.7)	0.076^†^
APOE ε4 carrier, *n* (%)	22 (18.2)	63 (30.7) b	67 (33.7) b	**0**.**010^†^**

Bold font represents statistical significance (*P* < 0.05) for group-wise tests by *one-way ANOVA or ^†^Pearson’s *χ*² tests.

CIND, cognitive impairment no dementia; NCI, no cognitive impairment; *n*, number of cases; SD, standard deviation.

a Significantly different from NCI (one-way ANOVA with *post hoc* Bonferroni test, *P*  *<* 0.05).

b Significantly different from NCI (Pearson’s *χ*², *P*  *<* 0.05).

c Significantly different from CIND (Pearson’s *χ*², *P*  *<* 0.05).

### Associations of serum ASC with vascular cognitive impairment and dementia

**Figure 1:**
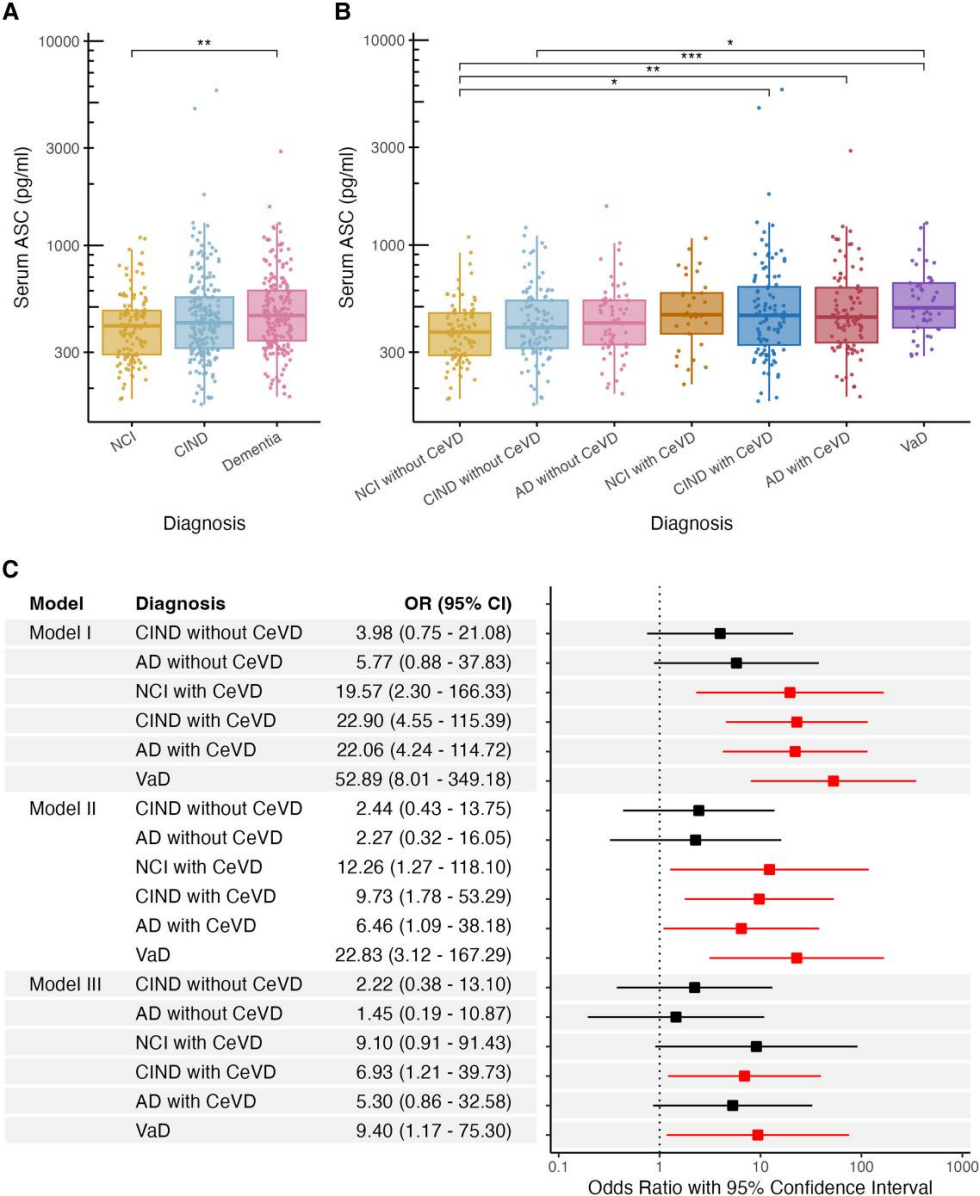
**Associations of serum ASC in with cognitive impairment and dementia.** Data are presented for serum ASC concentrations (**A**) before and (**B**) after segregation of diagnostic groups by the absence or presence of significant cerebrovascular disease (*n* & 531). Each data point represents serum ASC concentrations in individuals. In each boxplot, box shows median values and interquartile range (IQR & Q3-Q1) whereas whiskers show 1.5*IQR outside the IQR (i.e. Q1—1.5*IQR and Q3 + 1.5*IQR). Multiple group comparisons were performed using Kruskal–Wallis with Dunn’s Bonferroni *post hoc* tests, *P < 0.05, **P < 0.01 and ***P < 0.001. (**C**) Forest plots represent effect measures in odds ratio (OR, points) and 95% confidence interval (CI, lines), corresponding to the data presented in the table (*n* & 531). Multinomial regression analyses were performed on log-transformed serum ASC concentrations, using different models: Model I, unadjusted; Model II, adjusted for age, gender, education and APOE ε4 carrier; Model III, adjusted for age, gender, education, APOE ε4 carrier, hypertension and diabetes. Significant associations with *P* < 0.05 were indicated in red in the forest plots. Interpretation: Significant OR > 1 indicates that each 10-fold increase in serum ASC concentrations (1 unit of log10[ASC]) was associated with OR times higher chance of getting the respective cognitive outcome. Abbreviations: CeVD, cerebrovascular disease; CIND, cognitive impairment no dementia; CI, confidence interval, NCI, no cognitive impairment; OR, odds ratio.

Figure 1A shows that serum ASC levels were significantly higher in dementia [median (interquartile range, IQR) = 454 (260) pg/mL] compared to NCI [403.5 (187.8) pg/mL, Kruskal–Wallis ANOVA with Dunn’s *post hoc* test, *P* < 0.01], with CIND having intermediate values [418.5 (246.5) pg/mL] not significantly different from either NCI or dementia. After further stratifying each diagnostic group based on the subjects’ CeVD status, serum ASC levels were found to be significantly higher in all VCI groups, namely, CIND with significant CeVD (CIND + CeVD), AD with significant CeVD (AD + CeVD) and VaD (Kruskal–Wallis ANOVA with Dunn’s *post hoc* tests, *P* < 0.05; Fig. 1B). As outliers were observed, sensitivity analyses were performed after removal of seven extreme outliers beyond three IQR below the 25th percentile and above the 75th percentile. Similar trends were observed where serum ASC levels were significantly higher in dementia, especially in AD + CeVD and VaD groups (Kruskal–Wallis ANOVA with Dunn’s *post hoc* tests, *P* < 0.05; Supplementary Fig. 3).

Multinomial regression analyses with adjustment for demographic characteristic including age, gender, education and APOE ε4 showed similar association between higher serum ASC and all VCI groups (Model II; Fig. 1C). After additional adjustments for vascular risk factors including hypertension and diabetes (Model III), serum ASC remained significantly associated with the CIND + CeVD [odds ratio (OR) & 6.93; 95% confidence interval (CI) & 1.21–39.73] as well as with VaD (OR & 9.40; 95% CI & 1.17–75.30) groups, but not with AD + CeVD (OR & 5.30; 95% CI & 0.86–32.58) as shown in Fig. 1C.

### Associations of serum ASC with MRI markers of CeVD

**Figure 2:**
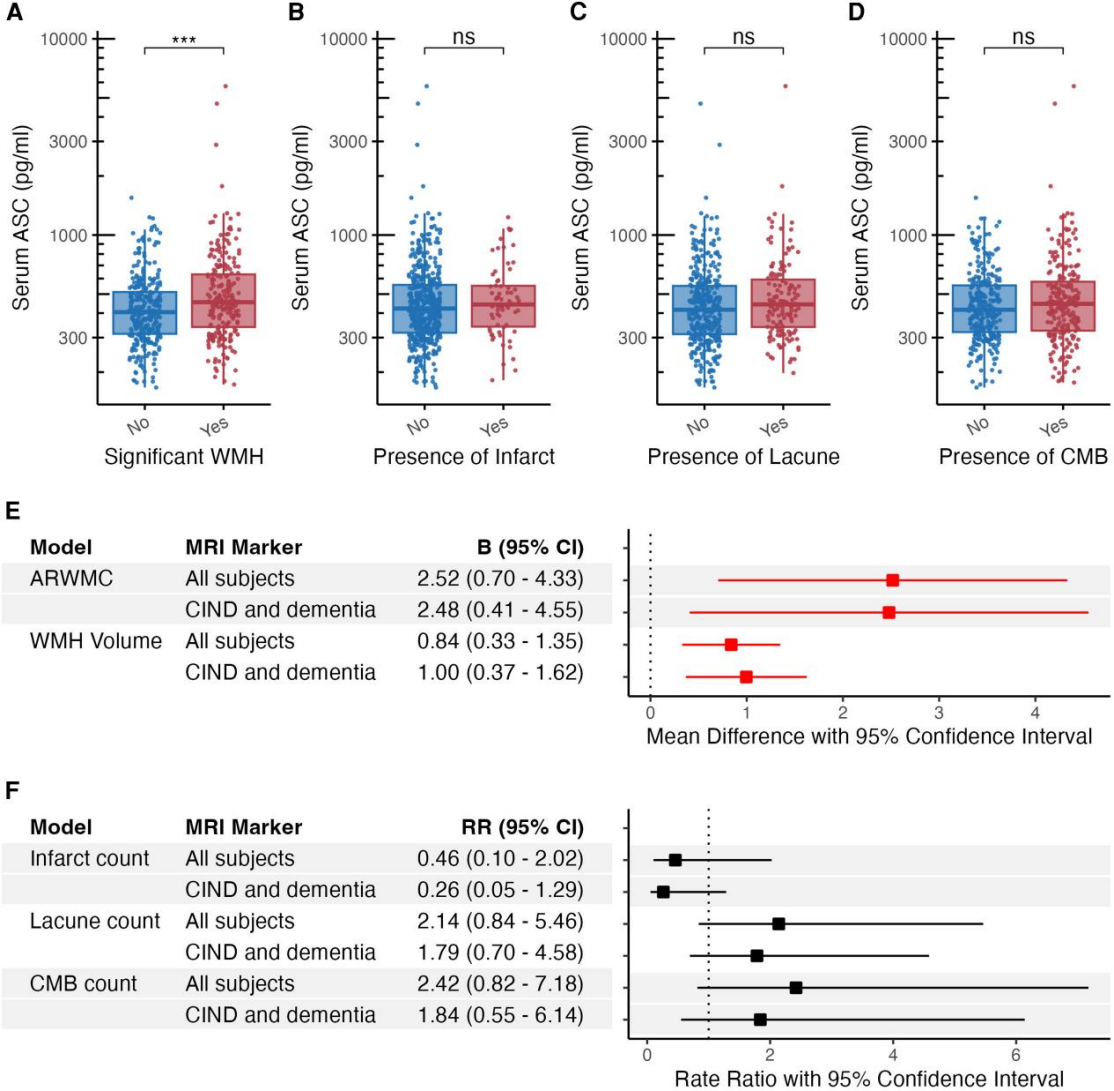
**Associations of serum ASC with MRI markers of cerebrovascular disease.** Data are presented for serum ASC concentrations (in pg/ml) in the absence (blue data points) and presence (red data points) of (**A**) white matter hyperintensities (WMH) defined by ARWMC score ≥ 8 (*n* = 530), (**B**) cortical infarct (*n* = 530), (**C**) lacune (*n* = 499) and (**D**) cerebral microbleed (CMB, *n* = 514). In each boxplot, box shows median and interquartile range (IQR = Q3-Q1), and whiskers show 1.5*IQR outside the IQR (i.e. Q1—1.5*IQR and Q3 + 1.5*IQR) of the distribution of data points (where each data point represents the ASC concentration of each participant classified under the respective groups). Group comparisons were performed using Mann–Whitney U-test, ***P < 0.001. Forest plots represent effect measures for (**E**) WMH defined by ARWMC score (*n* = 530), WMH volume expressed as percentage of total intracranial volume (*n* = 507), in mean difference (B, points) and 95% confidence interval (CI, lines), and for **(F)** infarct (*n* = 530), lacune (*n* = 499) and CMB (*n* = 514) counts in rate ratio (RR, points) and 95% CI (lines), corresponding to the data presented in table. Linear regression (for WMH) or zero-inflated negative binomial regression (for infarct, lacune and CMB counts) analyses were performed on log-transformed serum ASC concentrations, with adjustment for age, gender and hypertension. Analyses were performed for all subjects and for patients with CIND and dementia only. Significant associations with *P* < 0.05 were indicated in red in the forest plots. Interpretation: Significant MD > 0 indicates that each 10-fold increase in serum ASC concentrations (1 unit of log10[ASC]) was associated with B unit increase in the respective WMH measures. Significant RR > 1 indicates that each 10-fold increase in serum ASC concentrations (1 unit of log10[ASC]) was associated with RR times increase in the counts of lacune or CMB. Abbreviations: ARWMC, age-related white matter changes; B, mean difference; CeVD, cerebrovascular disease; CIND, cognitive impairment no dementia; CI, confidence interval; CMB, cerebral microbleed; NCI, no cognitive impairment; RR, rate ratio; WMH, white matter hyperintensities.

Since significant CeVD in VCI may be defined by multiple CeVD types,^24^ we subsequently assessed ASC in participant subgroups based on visually graded MRI markers. These included (i) significant WMH (ARWMC scores ≥ 8) and the presence of (ii) cortical infarcts, (iii) lacunes or (iv) cerebral microbleeds. Figure 2A shows that serum ASC concentrations were increased specifically in participants with significant WMH (Mann–Whitney U-test, *P* < 0.001), as no changes in serum ASC were observed in participants with cortical infarct, lacunes or cerebral microbleeds (Mann–Whitney U-tests, *P* > 0.05, Fig. 2B–D). We corroborated the findings from dichotomized variables by using regression analyses (adjusted for age, gender and hypertension) of CeVD continuous variables, as Fig. 2E showed that every 10-fold increase in serum ASC levels was significantly associated with WMH, defined by both increased ARWMC scores (B = 2.52; 95% CI = 0.70–4.33) and AccuBrain®-derived WMH volume (expressed as percentage of total intracranial volume, B = 0.84; 95% CI = 0.33–1.35). These associations remained significant even when the analyses were restricted to the cognitively impaired groups (i.e. CIND and dementia, see Fig. 2E). In contrast, no significant associations were observed with cortical infarct count (RR = 0.46; 95% CI = 0.11–2), lacune count (RR = 2.11; 95% CI = 0.83–5.37) and CMB count (RR = 2.24; 95% CI = 0.82–7.17, see Fig. 2F).

### Mediation effect of WMH on the association between serum ASC and VCI

**Figure 3:**
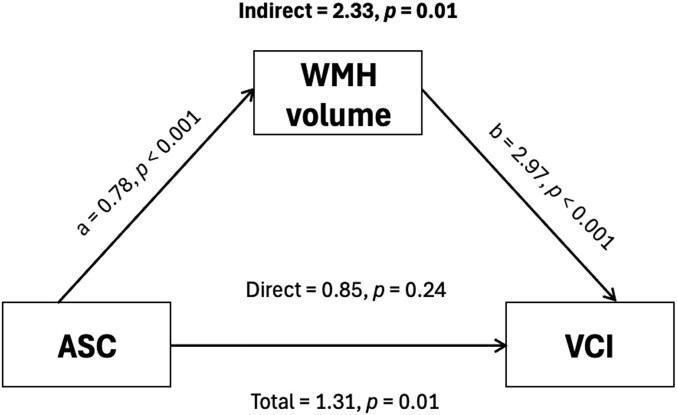
**Mediation effect of WMH volume on the associations between serum ASC and VCI.** Mediation effects of WMH volume expressed as percentage of total intracranial volume are reported as indirect effect, as estimated by the products of the direct effect of serum ASC on each CeVD marker (path a) and indirect effect of these markers on VCI (path b), i.e. a*b. The direct effect of serum ASC on VCI was estimated by adjusting the association between serum ASC and VCI (total effect) for the mediator (i.e. WMH volume). Pathway analysis were performed using generalized linear models, adjusted by age, gender, education, APOE ε4 status, hypertension and diabetes (*n* = 491). Abbreviations: VCI, vascular cognitive impairment; WMH, white matter hyperintensities.

Given that higher serum ASC was associated specifically with WMH, we performed causal mediation analyses to assess the potential mediation effect of WMH in the association between serum ASC and VCI. Using a counterfactual framework, the average causal mediation effect (ACME) was 0.082 (95% CI: 0.028–0.160, *P* & 0.005), representing an 8.2% increase in VCI probability via WMH. The proportion mediated was 73.3% (95% CI: 35.8% to 137%, *P* & 0.008), suggesting a strong mediation effect by WMH. Effect estimates expressed in log-odd scale are presented in Fig. 3, showing that the associations between ASC and VCI can largely be explainable by ASC effects on WMH severity.

## Discussion

While AD remains the commonest cause of dementia in the elderly, VCI may contribute significantly to the dementia burden, particularly in Asian populations which have high baseline burden of CeVD and vascular risk factors.^40-42^ Therefore, VCI represents an unmet research need for improved characterization and biomarker discovery alongside AD. In the present Singapore-based memory clinic cohort study, we investigated potential involvement of inflammasomes in AD and VCI by measuring serum levels of ASC. We selected ASC as it is specifically associated with inflammasome activation, but is also the common adaptor protein for three of the four canonical inflammasome sensors (AIM2, NLRP1, NLRP3)^17^ and can regulate the fourth sensor (NLRC4).^43^ Therefore, ASC is suitable as a general indicator of inflammasome activation. As far as we know, there is as yet no clinical study investigating biofluid-based inflammasome markers in VCI. One previous study, employing the same microfluidics immunoassay system used in the current work, reported elevated serum ASC concentrations in patients with mild cognitive impairment.^19^ Another study utilized single-molecule pull-down technology and identified increased ASC specks in both serum and cerebrospinal fluid of patients with AD and Parkinson’s disease.^20^ However, CeVD status was not investigated in either of the above-mentioned studies. Here, we found that compared to healthy controls, serum ASC was elevated in all VCI subgroups, including CIND + CeVD, AD + CeVD and VaD. Furthermore, of the various CeVD markers investigated, ASC was specifically associated with WMH severity (both visual rated ARWMC scores and automated volumetric quantitation), a neuroimaging biomarker of white matter lesions. Mediation analyses further demonstrated that the association between ASC and VCI can be largely explainable by ASC’s effects on WMH volume.

**Figure 4:**
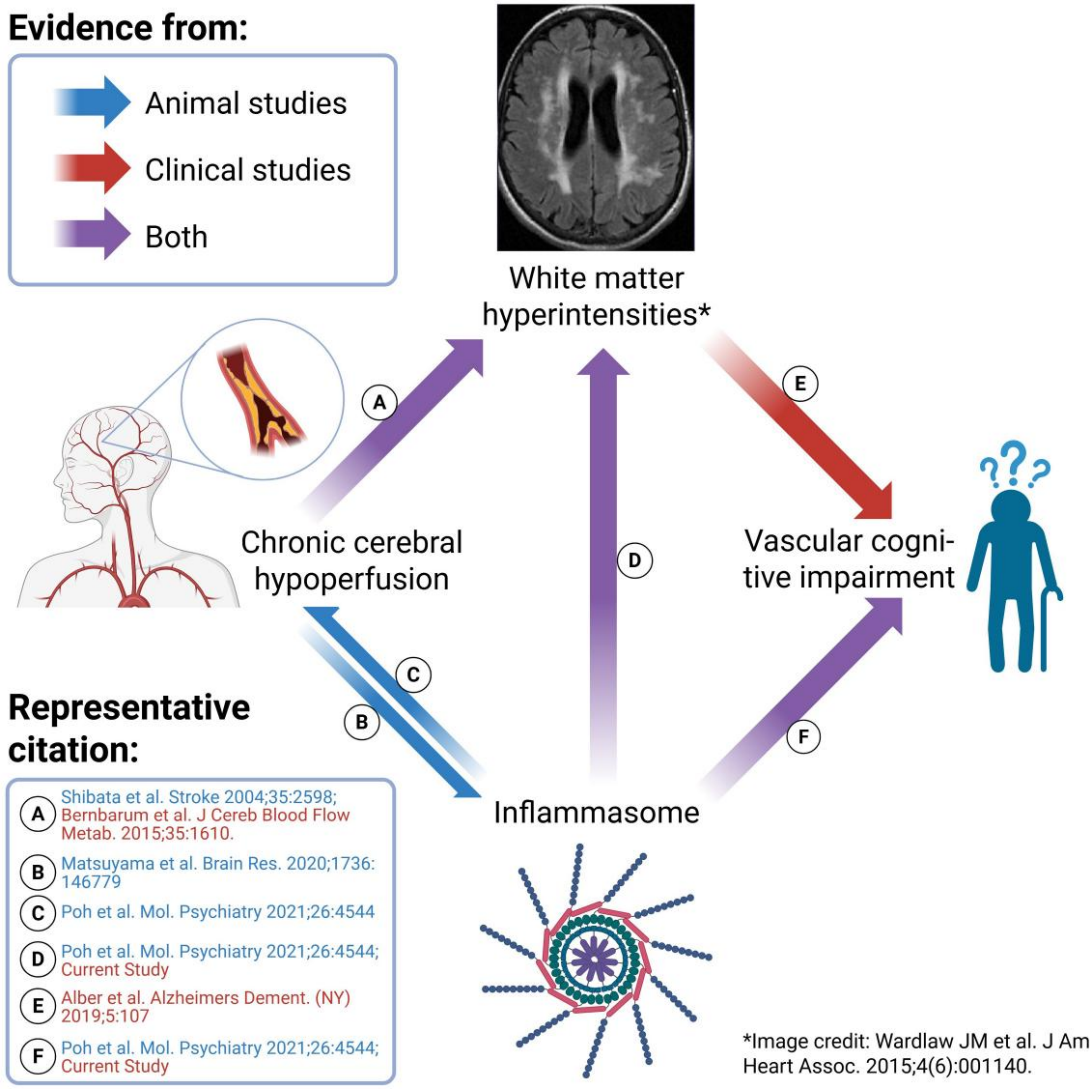
**Summary of current preclinical and clinical evidence for a putative role of inflammasome activation in VCI.** Schematic summary of current evidence of the putative causal links among chronic cerebral hypoperfusion (CCH), white matter hyperintensities (WMH), vascular cognitive impairment (VCI) and inflammasome activation, where available data from preclinical animal studies are denoted by blue arrows (citations in blue font), data from clinical studies denoted by red arrow (citations in red font) and data from both study types denoted by purple arrows. Therefore, CCH may lead to both (**A**) WMH and (**B**) inflammasome activation, with the latter in turn exacerbating CCH (**C**). Dysregulated activation of inflammasomes may (**D**)* lead to more severe WMH, and (**F**)* is associated with VCI, while (**E**) WMHs are also clinically known to contribute to VCI. Note that data from inflammasome activation is not limited to any specific sensor and in our study is indicated by the non-specific adaptor ASC. Figure created in BioRender: https://BioRender.com/ls7sufj. *Novel findings from the current study.

WMH, as a neuroimaging marker of white matter lesions (WML), is the commonest form of small vessel CeVD and is strongly associated with VCI.^44,45^ WMHs are thought to arise from multiple pathological processes including variable myelin and axonal loss, reactive gliosis, lipohyalinosis and arteriosclerosis in periventricular, subcortical or corpus callosum white matter regions.^46^ The current study adds elevated inflammasome activation as a correlate of WMH and, taken together with our mediation analysis (Fig. 3), provides support for a role of inflammasome dysregulation in the pathogenesis of WMH and VCI in the clinical setting. Work from our group and others have proposed chronic cerebral hypoperfusion (CCH) as the major underlying mechanism for VCI and associated CeVDs including WMH.^15,46^ Interestingly, there is also preclinical evidence that CCH may drive inflammasome activation,^16,21,23,47^ and while direct evidence in a CCH-associated WML model is not yet available, there are reports of small molecule inflammasome inhibitors ameliorating white matter disease-relevant processes like demyelination and white matter microstructural damage.^48,49^ Collectively, these lines of evidence indicate a central role for inflammasome activation within the interconnected associations between CCH, VCI and WMH (see Fig. 4). Importantly, because WMH can both progress and regress in a longitudinal clinical setting, with progression associated with cognitive decline and regression associated with preservation of cognition,^45,50^ our findings not only suggest the biomarker utility of inflammasome proteins like ASC for WMH, but also support inflammasome activation as a potential disease-modifying therapeutic target in VCI.

### Study limitations and future work

This study has several limitations. First, one reason we were able to uncover novel associations between serum ASC and WMH may be due to our specific cohort with relatively high baseline CeVD burden, and follow-up studies are needed to assess whether these findings may be applicable to other populations with differing vascular risk profiles. Second, the cross-sectional analysis of associations between serum ASC and neuroimaging markers precludes inferences about the temporal relationship between ASC levels and the progression of WMH and other CeVDs, necessitating follow-up investigations at multiple time points. Third, as previously explained, we selected ASC as the marker for inflammasome activation as it is a universal adaptor protein for multiple canonical inflammasome sensors. However, now that we have confirmation of serum ASC changes in VCI, further studies are needed to determine which of the upstream sensors are correlated with ASC. For example, preclinical and clinical evidence supports further investigations of AIM2 and NLRP3-related pathways.^16,51^ Similarly, downstream effectors of inflammasome activation like caspase-1, IL-1β and IL-18, some of which have previously been measured in AD or VaD,^16,52^ should be correlated with ASC to better characterize the mechanisms underlying inflammasome’s effect on WML. Fourth, although peripheral ASC levels have been interpreted as a marker of inflammasome activation in several clinical studies,^19,53,54^ it is not clear how well peripheral ASC correlates with speck formation, and further studies to directly measure specks (as has previously been reported^20^) are needed to confirm current findings. Last, while our findings of ASC increases were largely consistent in the VCI groups, hypertension and diabetes mellitus may confound ASC associations in certain VCI groups but not others, as these factors (Model III) resulted in loss of significance at multivariate analysis stage for AD + CeVD, but not for CIND + CeVD and VaD (see Fig. 1C). This is unsurprising as hypertension and diabetes are well established systemic vascular risk factors which have also been linked *a priori* to CeVDs such as WMH.^55,56^ However, our findings suggest that CeVD-specific and systemic vascular factors may influence different VCI subgroups to varying extents, highlighting the need for follow-up studies to further explore these complex relationships.

## Conclusions

Our findings highlight a potential role of inflammasome activation (as indicated by serum ASC changes) in VCI, specifically those with significant WMH. Follow-up studies are needed to validate current findings and further elucidate the underlying pathways and to assess the inflammasome system as biomarker and therapeutic target for VCI.

## Supplementary Material

fcag068_Supplementary_Data

## Data Availability

The data that support the findings of this study are available from the corresponding author upon reasonable request.
