# Supplementary material for: Elevated serum inflammasome adaptor protein ASC is associated with white matter hyperintensities in vascular cognitive impairment
Source: Brain Commun. 2026 Mar 5;8(2):fcag068. doi: 10.1093/braincomms/fcag068 (PMC13012222; doi:10.1093/braincomms/fcag068)
Supplement: fcag068_Supplementary_Data [file fcag068_supplementary_data.pdf]

Chai *et al*., Elevated serum inflammasome adaptor protein ASC is associated with white matter hyperintensities in vascular cognitive impairment

Supplementary Materials

Supplementary Table 1. Summary of Neuropsychological Battery and Component Tests

Cognitive Domain	Component Test(s)
**Executive Function:**	Frontal Assessment Battery [1]
**Attention:**	Digit Span, Visual Memory Span [2] and Auditory Detection [3]
**Language:**	Modified Boston Naming Test [4] and Verbal Fluency [5]
**Visuomotor Speed:**	Symbol Digit Modality Test [6, 7], Maze Task [7] and Digit Cancellation [8]
**Visuoconstruction:**	Weschler Memory Scale – Revised (WMS-R) Visual Reproduction Copy task [2], Clock Drawing [9] and Weschler Adult Intelligence Scale – Revised (WAIS-R) subtest of Block Design [10]
**Visual Memory:**	Picture Recall & Recognition Tasks, and WMS-R Visual Reproduction Recall & Recognition Task [2]
**Verbal Memory:**	Word List Recall & Recognition Tasks [11] and Story Recall Task

Supplementary Table 2. Cross-sectional demographic and disease characteristics of study cohort

	NCI without CeVD	NCI with CeVD	CIND without CeVD	CIND with CeVD	AD without CeVD	AD with CeVD	VaD	*p*-value
**n**	89	33	102	106	60	94	45	
**Age, years, mean (SD)**	68.5 (7.1)	72.2 (7.8)	73.0 (7.6)	75.1 (7.1)	74.7 (7.8)	77.6 (7.0)	74.0 (9.1)	**<0.001***
**Female, n (%)**	58 (64.6)	10 (30.3)	53 (52.0)	52 (49.1)	40 (66.7)	65 (68.4)	14 (31.1)	**<0.001**†
**Education ≤ elementary, n (%)**	27 (30.0)	12 (36.4)	34 (33.3)	60 (56.6)	40 (66.7)	72 (75.8)	31 (68.9)	**<0.001**†
**Hypertension, n (%)**	49 (54.4)	28 (84.8)	60 (60.0)	83 (78.3)	44 (73.3)	69 (73.4)	45 (100)	**<0.001**†
**Diabetes, n (%)**	21 (23.3)	8 (24.2)	38 (37.3)	31 (29.2)	24 (40.0)	34 (35.8)	24 (53.3)	**0.013**†
**Hyperlipidemia, n (%)**	60 (66.7)	27 (81.8)	78 (76.5)	80 (76.2)	41 (68.3)	70 (73.7)	39 (86.7)	0.176
**Heart disease, n (%)**	4 (4.5)	4 (12.1)	13 (12.7)	18 (17.0)	6 (10.2)	8 (8.4)	9 (20.9)	0.061
**APOE ε4 carrier, n (%)**	20 (22.2)	2 (6.5)	34 (34.0)	29 (27.6)	25 (41.7)	30 (31.9)	12 (26.7)	**0.013**†

AD = Alzheimer's disease, CeVD = cerebrovascular disease, CIND = cognitive impairment no dementia, NCI = no cognitive impairment, *n* = number of cases, SD = standard deviation, VaD = vascular dementia

Bold font represents statistical significance ($p < 0.05$) for group-wise tests by *One-way ANOVA or † Pearson's Chi-square tests.

Supplementary Table 3. Cross-sectional characteristics and serum ASC concentrations in CeVD subgroups

	Without CeVD	With CeVD	*p*-value
n	252	278	
**Age, years, mean (SD)**	71.8 (7.9)	75.4 (7.7)	**<0.001***
**Female, n (%)**	151 (59.9)	140 (50.4)	**0.027†**
**Hypertension, n (%)**	153 (61.2)	224 (80.9)	**<0.001†**
**Diabetes, n (%)**	83 (32.9)	97 (34.9)	0.635†
**Hyperlipidemia, n (%)**	179 (71)	215 (77.6)	0.083†
**Heart disease, n (%)**	23 (9.2)	38 (13.8)	0.102†
**APOE ε4 carrier, n (%)**	79 (31.6)	73 (26.6)	0.212†
**Cognitively impairment**			**<0.001†**
**CIND, n (%)**	102 (40.5)	106 (38.1)	
**Dementia, n (%)**	60 (23.8)	139 (50.0)	
**Serum ASC, pg/ml, median (IQR)**	390.5 (189.8)	455.5 (286.5)	**<0.001‡**

ASC = apoptosis-associated speck like protein containing a CARD, CeVD = cerebrovascular disease, CIND = cognitive impairment no dementia, IQR = interquartile range, NCI = no cognitive impairment, *n* = number of cases, SD = standard deviation

Bold fonts represent statistical significance ($p < 0.05$) by *Student t-test, † Pearson's Chi-square test or ‡Mann-Whitney U test.

Supplementary Figure 1. Participant recruitment flowchart

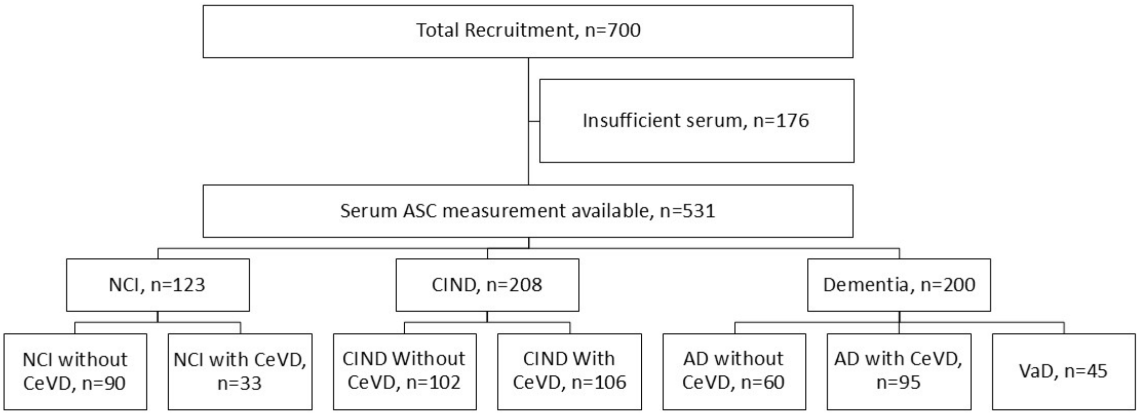

Supplementary Figure 1. A total of 700 participants were recruited, of which serum ASC measurements were available in 531 participants. Participants were clinically diagnosed as no cognitive impairment (NCI), cognitively impaired no dementia (CIND) and dementia (either Alzheimer's disease [AD] or vascular dementia [VaD]). Participants can be further stratified by the absence of presence of cerebrovascular disease (CeVD).

Supplementary Figure 2. Prevalence of abnormal brain amyloid burden determined by plasma p-tau217 levels in cognitive impairment and dementia subgroups

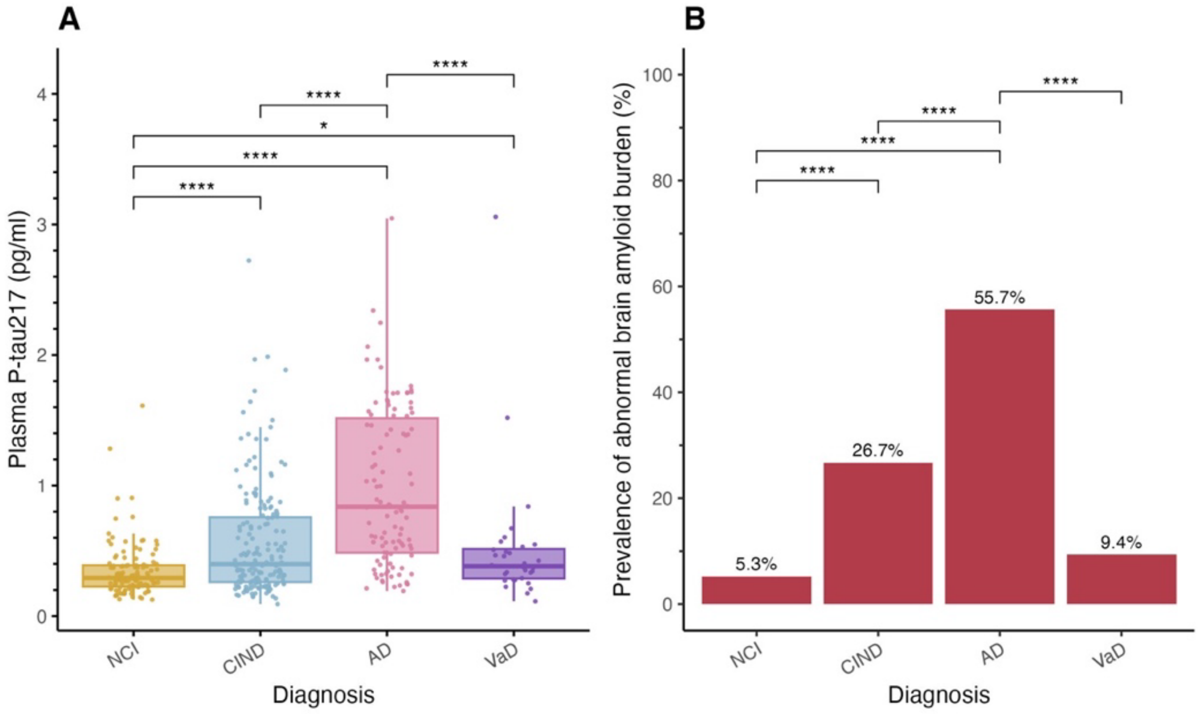

Supplementary Figure 2. (A) Data are presented for plasma tau phosphorylated at threonine 217 (p-tau217) concentrations measured using the SIMOA platform in each diagnostic group (n = 428, see Chong et al. [12] for details of the biomarker measurement). Each data point represents plasma p-tau217 concentrations in individuals. In each boxplot, box shows median values and interquartile range (IQR = Q3-Q1) whereas whiskers show 1.5*IQR outside the IQR (i.e. Q1 - 1.5*IQR and Q3 + 1.5*IQR). **(B)** Bar graphs are presented for the prevalence of abnormal brain amyloid burden determined by high plasma p-tau217 levels in each diagnostic groups (n = 428). Multiple group comparisons were performed using Kruskal Wallis with Dunn's Bonferroni *post-hoc* tests, *p<0.05 and ****p<0.0001.

Abbreviations: AD, Alzheimer's disease; CIND, cognitive impairment no dementia; NCI, no cognitive impairment; P-tau217, plasma tau phosphorylated at threonine 217; VaD, vascular dementia.

Supplementary Figure 3. Serum ASC levels in with cognitive impairment and dementia, after outlier removal

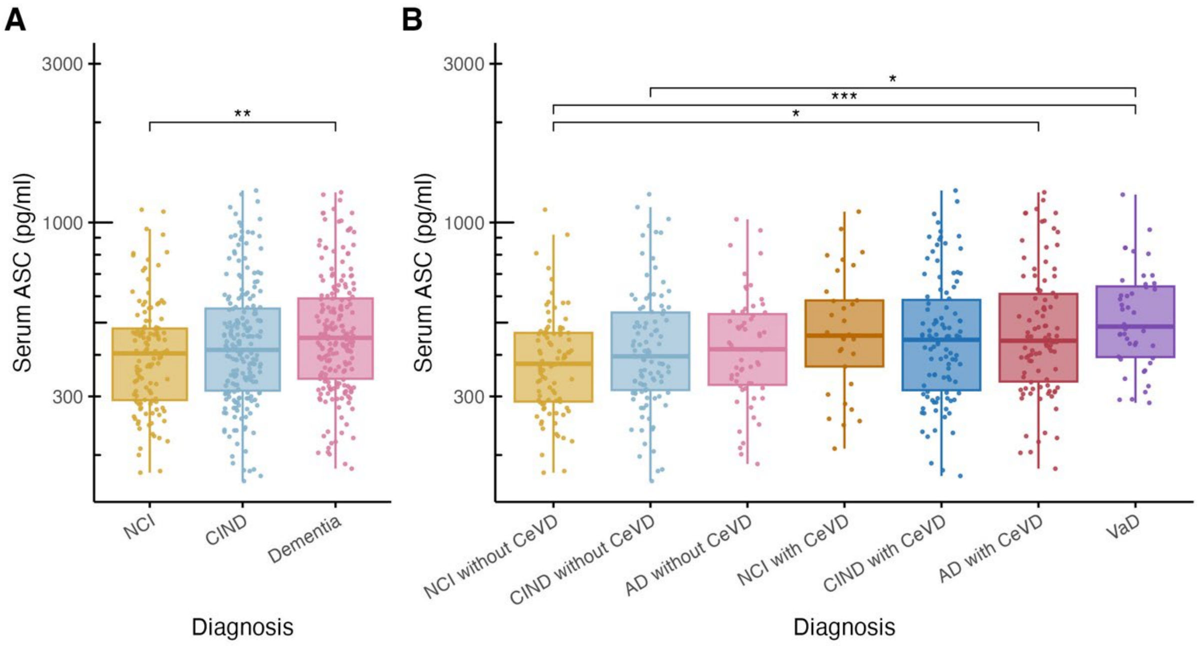

Supplementary Figure 3. Data are presented for serum ASC concentrations **(A)** before and **(B)** after segregation of diagnostic groups by the absence or presence of significant cerebrovascular disease, after outlier removal (n = 524). Each data point represents serum ASC concentrations in individuals.n each boxplot, box shows median values and interquartile range (IQR = Q3-Q1) whereas whiskers show 1.5*IQR outside the IQR (i.e. Q1 - 1.5*IQR and Q3 + 1.5*IQR). Multiple group comparisons were performed using Kruskal Wallis with Dunn's Bonferroni *post-hoc* tests, *p<0.05, **p<0.01 and ***p<0.001.

Supplementary References

1. Dubois B, Slachevsky A, Litvan I, Pillon B. The FAB: a Frontal Assessment Battery at bedside. Neurology. 2000;55(11):1621-6.

2. Wechsler D. Wechsler Memory Scale-Revised. 3rd ed. San Antonio, TX: Jovanovich; 1997.

3. Lewis RF, Rennick PM. Manual for the Repeatable Cognitive Percpetual-Motor Battery. Clinton Township, MI: Axon; 1979.

4. Mack WJ, Freed DM, Williams BW, Henderson VW. Boston Naming Test: shortened versions for use in Alzheimer's disease. J Gerontol. 1992;47(3):P154-8.

5. Isaacs B, Kennie AT. The Set test as an aid to the detection of dementia in old people. The British Journal of Psychiatry: The Journal of Mental Science. 1973;123(575):467-70.

6. Smith A. Symbol Digit Modalities Test. Los Angeles, 1973.

7. Porteus SD. The Maze Test and Clinical Psychology. Palo Alto, CA: Pacific Books; 1959.

8. Diller L, Ben-Yishay Y, Gerstman LJ. Studies in Cognition and Rehabilitation in Hemiplegia. New York: New York University Medical Center Institute of Rehabilitation Medicine; 1974.

9. Sunderland T, Hill JL, Mellow AM, Lawlor BA, Gundersheimer J, Newhouse PA, et al. Clock drawing in Alzheimer's disease. A novel measure of dementia severity. J Am Geriatr Soc. 1989;37(8):725-9.

10. Wechsler D. Wechsler Adult Intelligence Scale-Revised. San Antonio, TX: Harcourt Brace Jovanovich; 1981.

11. Sahadevan S, Tan NJ, Tan T, Tan S. Cognitive testing of elderly Chinese people in Singapore: influence of education and age on normative scores. Age and Ageing. 1997;26(6):481-6.

12. Chong, J.R., et al., Clinical utility of plasma p-tau217 in identifying abnormal brain amyloid burden in an Asian cohort with high prevalence of concomitant cerebrovascular disease. Alzheimers Dement, 2025;21(2): e14502.
